# Diversity and Dynamics of Bacterial Communities in the Digestive and Excretory Systems across the Life Cycle of Leafhopper, *Recilia dorsalis*

**DOI:** 10.3390/insects14060545

**Published:** 2023-06-12

**Authors:** Qiuyan Huang, Hong-Wei Shan, Jian-Ping Chen, Wei Wu

**Affiliations:** State Key Laboratory for Managing Biotic and Chemical Threats to the Quality and Safety of Agro-Products, Key Laboratory of Biotechnology in Plant Protection of Ministry of Agriculture and Zhejiang Province, Institute of Plant Virology, Ningbo University, Ningbo 315211, China; nbuhqy@foxmail.com (Q.H.); shanhongwei@nbu.edu.cn (H.-W.S.)

**Keywords:** *Recilia dorsalis*, leafhopper, microbiome, 16S rRNA, developmental stage

## Abstract

**Simple Summary:**

*Recilia dorsalis* is a destructive insect pest in rice-producing regions of Asia. It causes enormous damage to rice crops by directly feeding on phloem-sap or transmitting various viral diseases. Like other insects, *R. dorsalis* harbors numerous symbiotic microorganisms that play important roles in host nutrition, adaptation, and stress resistance. To understand the interactions between microorganisms and their host insects, we analyzed the bacterial community structure and dynamics of the digestive and excretory systems of *R. dorsalis* at different developmental stages using high-throughput sequencing. We investigated the initial source of microorganisms in *R. dorsalis* and compared the bacterial community structure and abundance of each tissue at different developmental stages. In addition, we conducted an analysis of the core bacterial community of *R. dorsalis* and predicted its function. Our findings enhance our comprehension of the interactions between insects and symbiotic microorganisms, which may facilitate the establishment of novel biological control techniques against *R. dorsalis*.

**Abstract:**

*Recilia dorsalis* is a notorious rice pest that harbors numerous symbiotic microorganisms. However, the structure and dynamics of bacterial communities in various tissues of *R. dorsalis* throughout its life cycle remain unclear. In this study, we used high-throughput sequencing technology to analyze the bacterial communities in the digestive, excretory, and reproductive systems of *R. dorsalis* at different developmental stages. The results showed that the initial microbiota in *R. dorsalis* mostly originated from vertical transmission via the ovaries. After the second-instar nymphs, the diversity of bacterial communities in the salivary gland and Malpighian tubules gradually decreased, while the midgut remained stable. Principal coordinate analysis revealed that the structure of bacterial communities in *R. dorsalis* was primarily influenced by the developmental stage, with minimal variation in bacterial species among different tissues but significant variation in bacterial abundance. *Tistrella* was the most abundant bacterial genus in most developmental stages, followed by *Pantoea*. The core bacterial community in *R. dorsalis* continuously enriched throughout development and contributed primarily to food digestion and nutrient supply. Overall, our study enriches our knowledge of the bacterial community associated with *R. dorsalis* and provides clues for developing potential biological control technologies against this rice pest.

## 1. Introduction

Insects, one of the most numerous, evolutionarily oldest, and widely distributed animal groups on Earth, have established various types of associations with microorganisms over the course of their long evolution [1,2]. Insects can establish a close symbiotic relationship with microorganisms that help them acclimate to fluctuating dietary conditions by providing crucial nutrients that are deficient in their host diet [3,4]. By augmenting the nutritional uptake of the host insect, symbiotic microorganisms can impact life parameters, including growth and development, lifespan, as well as mating and reproduction [3,4,5,6,7]. Additionally, symbiotic microorganisms are responsible for regulating the host insect’s resistance to different adversities and harmful biological factors, including detoxifying exogenous biotoxins, regulating the host’s pesticide resistance, protecting it from pathogens, and influencing the efficiency of pathogen transmission through vector insects [8,9,10,11,12,13,14]. Thus, symbiotic microorganisms play a crucial role in the successful evolution of insects, serving as important drivers of insect diversity and adaptation [2,4,15,16].

Increasing evidence indicates that the health and adaptation of host insects are closely tied to the diversity and homeostasis of their microbial communities. Symbiotic microorganisms can be classified into obligate and facultative symbionts based on their degree of interdependence with host insects [16]. Obligate symbionts are integral to the survival of their insect host by producing important amino acids and scarce vitamins to supplement the nutritional needs of their host. Obligate symbionts are located in specific tissues and cells within the insect host and can be vertically transmitted to offspring [17,18]. Obligate symbionts are stable within the host, and their abundance is related only to the insect’s developmental stage [17,18]. Most symbiotic microorganisms in insects are facultative symbionts that are non-essential for insect survival but crucial for nutrient utilization, resistance, and fitness [19,20]. Facultative symbionts do not have a fixed location in the host insects and exhibit dynamic population structures depending on the dietary regimes, host environments, and developmental stages of the host [20,21].

The developmental stage of insects exerts a significant influence on the symbiotic microorganism community structure within them [22]. In mosquitoes, the microorganisms in their bodies steadily increase during the larval stage and peak in the last instar, just before the last defecation. Microorganism abundance increases again in the pupal stage but is found to be very low in adult mosquitoes [23,24]. In contrast, *Drosophila* shows no significant change in symbiotic diversity over its life cycle, but abundance varies. *Lactobacillus* is dominant in young *Drosophila* flies, and *Acetobacteria* is dominant in older flies [25]. In some Lepidoptera species, such as *Spodoptera littoralis* and *Grapholita molesta*, microbial diversity varies in a “U” pattern during development, reaching a minimum in pupae [26,27]. In several Hemiptera, microbial composition and abundance exhibit diverse changes during insect development. In *Pyrrhocoris apterus*, microbial diversity decreases until the third instar and remains stable thereafter [28]. In multiple triatomines, microbial diversity gradually decreases with insect development [29]. In *Nilaparvata lugens*, microbial diversity remains stable throughout the life cycle [30].

*Recilia dorsalis* (Cicadellidae: Deltocephalinae), a destructive insect pest in rice-producing regions of Asia, causes enormous damage to rice crops by directly feeding on phloem-sap or transmitting various viral diseases [31,32]. Leafhoppers establish symbiotic relationships with “*Candidatus* Sulcia muelleri” (hereafter *Sulcia*) and a proteobacterium partner, e.g., “Candidatus Nasuia deltocephalinicola” (hereafter *Nasuia*), “*Candidatus* Baumannia cicadellinicola”, “*Candidatus* Zinderia insecticola”, “*Candidatus* Vidania fulgoroideae”, “*Candidatus* Hodgkinia cicadicola” [33]. These symbiotic bacteria provide the leafhopper host with ten essential amino acids that are not present in their diet [33]. In addition to obligate symbionts, leafhoppers also harbor various facultative symbionts, including *Wolbachia*, *Rickettsia* and *Cardinium*, which confer fitness benefits to the insect host, such as enhanced growth rate, body size, and reproductive capacity [34,35,36,37]. However, there are a lack of studies that investigate the dynamics of bacterial communities in *R. dorsalis* during various developmental periods and in different tissues, as well as the migration patterns and roles of the core microbiota. This study employed high-throughput amplicon sequencing to investigate the bacterial communities associated with *R. dorsalis* throughout its developmental stages and various tissues. The purpose of this study was to characterize the bacterial community of *R. dorsalis*, elucidate the dynamics of microbiota during *R. dorsalis* development, and establish the core bacterial community that persists across the entire *R. dorsalis* life cycle. The KEGG orthology (KO) pathway of core bacteria was also predicted. The research findings presented in this study have the potential to advance the understanding of electrifying host–microbe interactions and microbe-mediated pest management, thus contributing to the development of future research in these areas.

## 2. Materials and Methods

### 2.1. Insect Rearing

Adult leafhoppers *Recilia dorsalis* were collected from a rice field in Jiaxing, Zhejiang Province, China in September 2020. The *R. dorsalis* were reared in an insect-proof greenhouse at a stable temperature of 26 ± 1 °C, with a 16:8 h light-to-dark cycle and 50 ± 5% relative humidity for over two years. TaiChung Native 1 (TN1) rice was grown under the same conditions of leafhopper-feeding. To collect leafhoppers at different developmental stages, approximately 1000 adult *R. dorsalis*, with a roughly equal sex ratio, were collected and allowed to oviposit on rice. The rice plants were replaced every 2 days. The newly hatched nymphs were considered first instars, and after molting the nymphs were considered second instars and so on. Fifth instar nymphs were transferred to a new insect-rearing cage, and newly emerged adults were collected every 24 h (1-day-emerged adults) and transferred to a new insect rearing cage for feeding.

### 2.2. Tissue Sample Collection

First- to fifth-instar nymphs and 7-day-emerged adults of *R. dorsalis* were used for sample preparation. To clear the allochthonous microorganisms in their digestive tracts, we subjected early-instar nymphs (first to third instars), late-instar nymphs (fourth to fifth instars), and adults to 6-, 12-, and 12-h starvation treatment, respectively. The insects were then subjected to surface sterilization with 75% ethanol for 90 s, followed by rinsing three times with sterilized deionized water. Consequently, salivary gland (Sg), midgut (Mg), Malpighian tube (Mt), ovary (Ov), and testis (Te) were dissected under a dissecting microscope using sterile needles and forceps in chilled phosphate buffer solution (pH 7.4; 140 mmol/L NaCl, 2.7 mmol/L KCl, 10 mmol/L Na_2_HPO_4_, 1.8 mmol/L KH_2_PO_4_). We collected twenty groups of tissues, each containing three biological replicates, resulting in a total of 60 tissue samples. To obtain each tissue sample, we dissected at least 100 leafhoppers. All tissue samples were immediately flash-frozen in liquid nitrogen and stored at −80 °C until DNA extraction.

### 2.3. DNA Extraction and PCR Amplification

The total DNA was extracted from each tissue sample using a DNeasy Tissue Kit (Qiagen, Hilden, Germany) following the manufacturer’s instructions. The quality and quantity of DNA were assessed using a Nanodrop 2000 spectrophotometer (Thermo Fisher Scientific, Waltham, MA, USA). Extracted DNA was stored at -80 °C until further use. Bacterial compositions in *R. dorsalis* were determined through PCR amplification of the V3–V4 region of the 16S rRNA gene using universal primers 338F (5′−barcode-ACTCCTACGGGAGGCAGCAG−3′) and 806R (5′−barcode-GGACTACHVGGGTWTCTAAT−3′) (synthesized in Songong BioTech, Shanghai, China). PCR amplification was performed using the Expand High Fidelity plus PCR system (Roche) according to the manufacturer’s instructions. The PCR amplifications were carried out in a total volume of 25 μL containing 25 ng template DNA, 2.5 μL of each forward and reverse primers, and 12.5 μL of Phusion^®^ Hot Start Flex 2X Master Mix (New England BioLabs, Ipswich, MA, USA). The following condition was used for each PCR reaction: an initial denaturation at 98 °C for 30 s, 32 cycles of denaturation at 98 °C for 10 s, annealing at 54 °C for 30 s, and extension at 72 °C for 45 s; and final extension at 72 °C for 10 min. After confirming the PCR products with 2% agarose gel electrophoresis, ultrapure water was employed as a negative control to exclude the possibility of false-positive PCR results. Subsequently, PCR products were purified using AMPure XT beads (Beckman Coulter Genomics, Danvers, MA, USA) and quantified by Qubit (Invitrogen, Waltham, MA, USA). The amplicon library size and quantity were assessed using the Agilent 2100 Bioanalyzer (Agilent, Santa Clara, CA, USA) and the Library Quantification Kit for Illumina (Kapa Biosciences, Woburn, MA, USA), respectively. Finally, the libraries were paired-end sequenced (2 × 250 bp) using the Illumina NovaSeq 6000 platform according to the manufacturer’s recommendations, provided by LC-Bio Technology Co., Ltd. (Hangzhou, China).

### 2.4. High-Throughput Sequencing and Analysis

Paired-end reads were assigned to samples based on their unique barcode and truncated by cutting off the barcode and primer sequence. Paired-end reads were merged using FLASH [38]. Quality filtering was performed on the raw reads under specific filtering conditions to obtain high-quality clean tags according to fqtrim (v0.94), and the chimeric sequences were filtered using Vsearch (v2.3.4) [39]. After dereplication using DADA2 [40], we obtained feature table and denoised feature sequences, which are called amplicon sequence variants (ASVs).

Alpha diversity and beta diversity were calculated by normalizing to the same sequences randomly. Then, according to the SILVA (release 138) classifier, feature abundance was normalized using the relative abundance of each sample [41]. Alpha diversity is applied in analyzing complexity of species diversity for a sample through four indices, including Chao1, Shannon, Simpson, Pielou-e, and all these indices in our samples were calculated with QIIME2 [42]. Beta diversity was calculated based on weighted and unweighted UniFrac distances in QIIME2, and the graphs were drawn by R package. Blast was used for sequence alignment, and the feature sequences were annotated with the SILVA database for each representative sequence. Other diagrams were implemented using the R package (v3.5.2).

Phylogenetic Investigation of Communities by Reconstruction of Unobserved States 2 (PICRUSt2) analysis (https://github.com/picrust/picrust2) [43] was used to predict the metagenome in the samples, and then the metagenome functions were predicted, and the data were exported into levels 1 and 2 of the Kyoto Encyclopedia of Genes and Genomes (KEGG) database pathways.

### 2.5. Statistical Analysis

Statistical analysis was performed using the software SPSS 20.0 (IBM Corporation, Armonk, NY, USA). Significant difference was analyzed by one-way analysis of variance (ANOVA) with means compared by applying the Fisher’s Least Significant Difference (LSD) test. Results are shown as the mean ± SEM. The level of significance and very significant for results was set at *p* < 0.05, respectively. Univariate analysis based on the relative abundances of genera was performed using the linear discriminant analysis effect size (LEfSe) method [44].

## 3. Results

### 3.1. General Characteristics of the Sequencing Data

To investigate the structure and dynamics of bacterial communities in *R. dorsalis* throughout all developmental stages, we collected a total of 60 tissue samples, including midgut, salivary gland, and Malpighian tubules from first-instar nymphs to adult leafhoppers, as well as testes and ovaries from adult leafhoppers. Subsequently, we extracted DNA from the samples, performed PCR amplification of the V3–V4 variable region of the 16S rRNA gene, and sequenced the samples using the Illumina NovaSeq platform.

**Figure 1:**
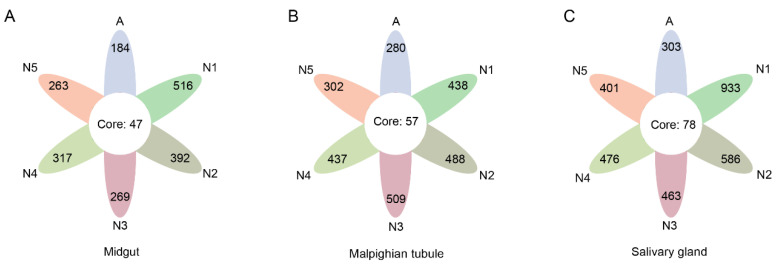
The flower plot diagram shows the number of core ASVs and ASVs specific to each individual class in the midgut (**A**), Malpighian tubule (**B**), and salivary glands (**C**) of leafhoppers *R. dorsalis* at different developmental stages. N1, N2, N3, N4, N5, A refer to 1st−instar nymphs, 2nd−instar nymphs, 3rd−instar nymphs, 4th−instar nymphs, 5th−instar nymphs, and adults, respectively.

A total of 5,026,342 raw tags were obtained from the high-throughput sequencing. After quality filtering and read merging, the sequencing of 60 samples generated 4,569,758 valid tags with an average of 76,163 valid tags per sample (Supplementary Table S1). A total of 3333 ASVs were identified, with a minimum of 99 ASV per sample and a maximum of 589 ASV per sample (Supplementary Tables S1 and S2). Core ASVs were identified in the midgut, Malpighian tubule, and salivary glands of leafhoppers at all developmental stages, with 47, 57, and 78 core ASVs, respectively (Figure 1). In addition, 12 ASVs present in all samples in different tissues of leafhopper *R. dorsalis* at different developmental stages (Supplementary Figure S1, Tables S1 and S2). The highest number of ASVs was found in first-instar nymphs, while the lowest number was found in adults (Supplementary Tables S1 and S2).

### 3.2. Diversity of Bacterial Communities in Different Tissues throughout the Life Cycle of R. dorsalis

**Figure 2:**
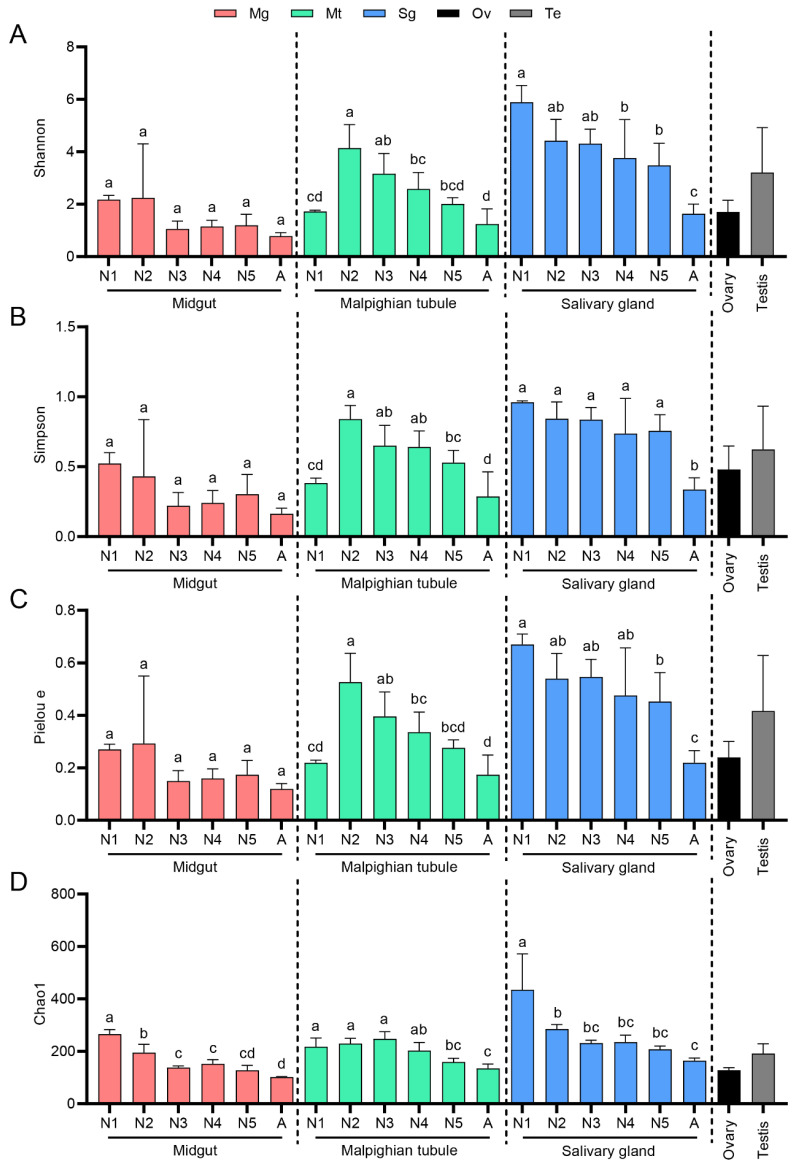
Alpha diversity among microbiota in the midgut, Malpighian tubule, salivary gland, ovary, and testis of the leafhopper *R. dorsalis* at different developmental stages. Shannon (**A**) and Simpson (**B**) indexes represent the species diversity, Pielou_e (**C**) index represents species evenness, Chao1 (**D**) species indexes represent species richness. Each bar represents the mean ± SEM of three replicates. Different lowercase letters indicate significant differences (ANOVA, *p* < 0.05). N1, N2, N3, N4, N5, A refer to 1st−instar nymphs, 2nd−instar nymphs, 3rd−instar nymphs, 4th−instar nymphs, 5th−instar nymphs, and adults, respectively.

We estimated the richness and diversity of bacterial communities at different developmental stages and in various tissues of *R. dorsalis* by alpha diversity indices, including Shannon, Simpson, Pielou_e, and Chao1 estimators (Supplementary Table S3). Bacterial Chao1 richness and Shannon diversity rarefaction curves were saturated with enhanced sequence numbers (Supplementary Figure S2), indicating that the sequencing depth was sufficient. When the samples were grouped according to their tissue types, the results of alpha diversity analysis showed that there was no significant difference in the bacterial community richness of the microbiota in the midgut at different developmental stages of insects (Figure 2). Malpighian tubules had the highest species richness in the second-instar nymphs, followed by third- to fifth-instar nymphs, and the lowest species richness in first-instar nymphs and adults. The salivary glands showed a continuous decrease in species richness gradually with leafhopper development (Figure 2). Furthermore, when samples were grouped according to the different developmental stages of *R. dorsalis*, bacterial community abundance followed a pattern of highest in salivary glands and lowest in midgut during most developmental stages of *R. dorsalis* (Supplementary Figure S3).

**Figure 3:**
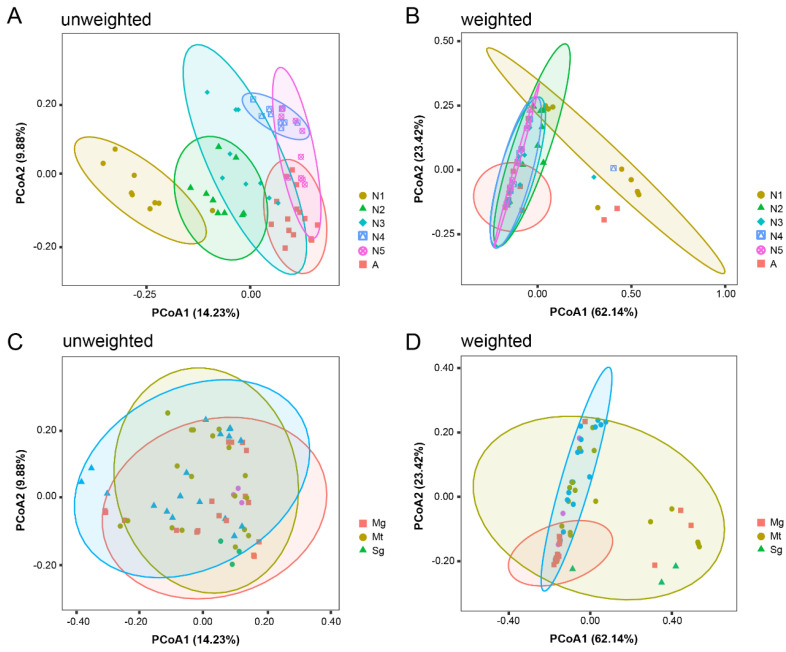
Beta diversity of microbiota in different tissues of the digestive and excretory systems of leafhoppers *R. dorsalis* at different developmental stages. PCoA ordination of unweighted (**A**) and weighted (**B**) UniFrac distances among different developmental stages of *R. dorsalis*. PCoA ordination of unweighted (**C**) and weighted (**D**) UniFrac distances among midgut, Malpighian tubule, and salivary gland of *R. dorsalis*. N1, N2, N3, N4, N5, A refer to 1st−instar nymphs, 2nd−instar nymphs, 3rd−instar nymphs, 4th−instar nymphs, 5th−instar nymphs, and adults, respectively. Mg, midgut; Mt, Malpighian tubule; Sg, salivary gland.

In order to evaluate the main driver of bacterial composition in the samples, we employed principal coordinates analysis (PCoA) and analysis of similarities (ANOSIM) with both unweighted and weighted UniFrac distances to compare bacterial β-diversity in *R. dorsalis* samples grouped by developmental stage or tissue type. PCoA of unweighted UniFrac distances revealed significant differences in bacterial composition among developmental stages but not among tissues (Figure 3A,C). Developmental stage was therefore the main driver of bacterial composition. PCoA analysis based on weighted UniFrac distances revealed a similar bacterial community composition in the midgut, Malpighian tubules, and salivary glands. However, there were significant differences in the abundance of bacterial communities. Furthermore, bacterial community composition varied significantly among developmental stages, with continued enrichment of the core bacterial community (Figure 3B,D).

### 3.3. Bacterial Community at Different Developmental Stages and in Various Tissues of R. dorsalis

**Figure 4:**
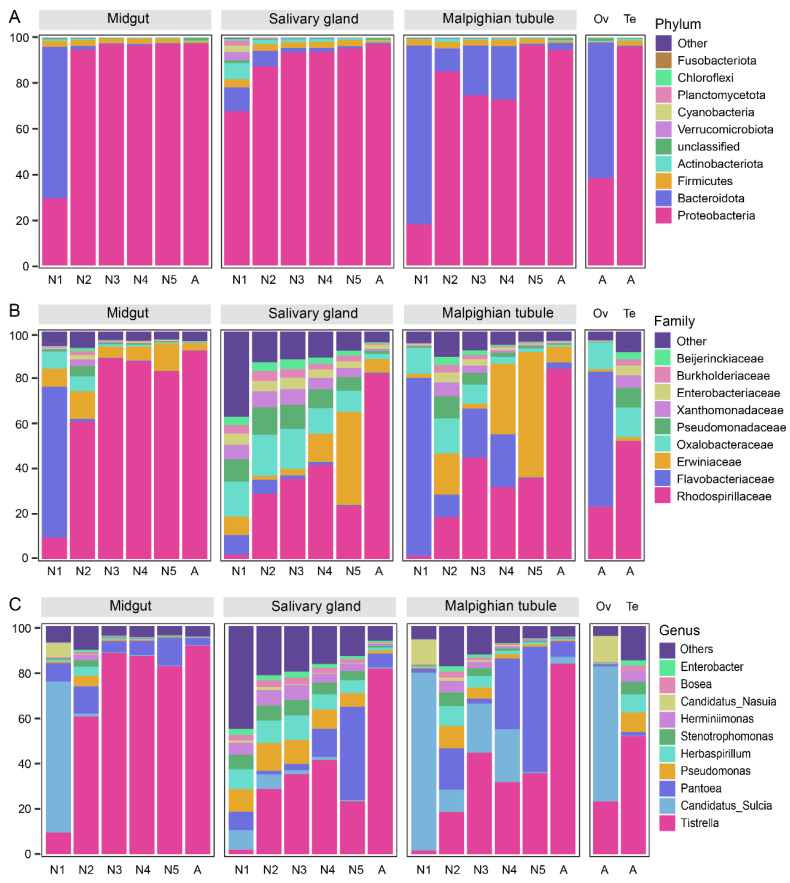
Bacterial community dynamics among in the midgut, salivary glands, Malpighian tubule, ovary, and testis of the leafhopper *R. dorsalis* at different developmental stages. The relative abundance of bacterial communities was depicted at the phylum (**A**), family (**B**), and *genus* (**C**) levels in *R. dorsalis*. Each data column represents the mean of three biological replicate samples, with different colors indicating distinct annotated information. Additionally, the term “others” denotes all species not annotated above. N1, N2, N3, N4, N5, A refer to 1st−instar nymphs, 2nd−instar nymphs, 3rd−instar nymphs, 4th−instar nymphs, 5th−instar nymphs, and adults, respectively.

A total of 3333 ASVs were annotated into 26 phyla, 67 classes, 165 orders, 327 families, 768 genera, and 1126 species (Supplementary Table S2). We performed in-depth analyses of bacterial communities that were among the top 10 in relative abundance at the phylum, family, and genus taxonomic level (Figure 4). At the phylum level, Proteobacteria was the dominant phylum in all tissues except for the ovaries and first instar nymphs, where Bacteroidetes was more abundant (Figure 4A). *Rhodospirillaceae*, *Erwiniaceae*, and *Oxalobacteraceae* were dominant at the family level (Figure 4B), and *Tistrella*, *Pantoea*, *Pseudomonas*, *Herbaspirillum*, and *Stenotrophomonas* were dominant at the genus level. *Sulcia* was the dominant genus in the midgut and Malpighian tubules of first-instar nymphs and ovaries (Figure 4C). *Tistrella* became increasingly dominant in the midgut, Malpighian tubules, and salivary glands with the continued development of *R. dorsalis*. The bacterial community structure in the midgut and Malpighian tubules of first-instar nymphs was similar to that in the ovaries of adults but significantly different from that in the testes, indicating that symbiotic microorganisms in *R. dorsalis* are mainly transmitted vertically through the ovaries (Figure 4). At the tissue level, the salivary gland had the highest bacterial diversity, while the midgut maintained a stable intestinal microbiota after the second-instar nymph. The diversity of bacterial communities in the Malpighian tubules decreased with insect development, and *Pantoea* was the dominant bacteria in some developmental stages (Figure 4).

### 3.4. Significantly Different Bacterial Communities at Different Developmental Stages and in Various Tissues of R. dorsalis

**Figure 5:**
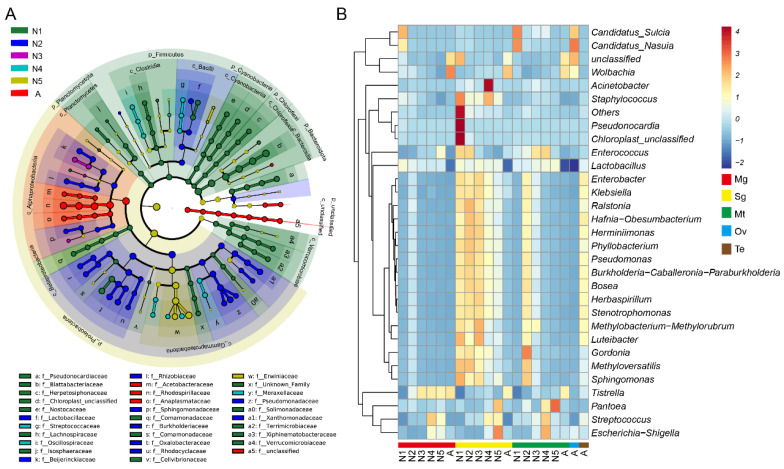
Significant difference analysis of the bacterial community in the development of *R. dorsalis*. (**A**) LEfSe analysis showing significant differences of bacterial species at the level of phylum, class, order, family, and genus from inside to outside. Different color nodes represent microbiota that is significantly enriched at the corresponding development stages. Each circle’s diameter was proportional to the taxonomic abundance. (**B**) Heat map of the top 30 genera in terms of relative abundance at the genus level. Samples are clustered according to the similarity between their constituents and arranged in horizontal order. The color scale gradients represent the log10-normalized values of abundances and symbolize variation of different species in the sample. N1, N2, N3, N4, N5, A refer to 1st−instar nymphs, 2nd−instar nymphs, 3rd−instar nymphs, 4th−instar nymphs, 5th−instar nymphs, and adults, respectively.

Linear discriminant analysis effect size was used to identify significant differences in bacteria at the phylum to genus level among life stages of *R. dorsalis*. Distinct microbiotas are enriched at each stage, from phylum to genus (Figure 5A, Supplementary Table S4). For instance, first-instar nymphs have significant enrichment of *Pseudonocardia*, *Terrimicrobium*, *Herpetosiphon*, *Xiphinematobacter*, *Isosphaera*, *Fontimonas*, *Sulcia*, *Lactobacillus*, *Aquamicrobium*, *Nasuia*, *Curvibacter*, and *Mucilaginibacter*, while second-instar nymphs have significant enrichment of *Ralstonia*, *Phyllobacterium*, *Pseudomonas*, *Stenotrophomonas*, *Lactobacillus*, *Herminiimonas*, *Herbaspirillum*, *Burkholderia*, *Megamonas*, *Bosea*, *Sphingomonas, Luteibacter,* and *Methyloversatilis*. Third-instar nymphs have significant enrichment of *Methylobacterium Methylorubrum, Sphingopyxis,* and *Aquamicrobium*, while fourth-instar nymphs have significant enrichment of *Acinetobacter, Streptococcus, Escherichia Shigella,* and *Pantoea*. Fifth-instar nymphs have enrichment of *Erysipelatoclostridium* and *Pantoea*. Adults have enrichment of *Wolbachia, Asaia, Tistrella, Parabacteroides*, and *Bifidobacterium* (Figure 5A, Supplementary Table S4).

We plotted a heatmap of the top 30 genera based on their relative abundance to investigate the abundance differences of significantly enriched bacteria in different tissues and developmental stages of *R. dorsalis*. *Sulcia* and *Nasuia* were enriched in the midgut and Malpighian tubules of first-instar nymphs and adult ovaries, respectively, while *Wolbachia* was enriched in adults (Figure 5B). *Tistralla* increased in abundance in the midgut with leafhopper development, and *Pantoea* was enriched in the Malpighian tubules and salivary glands of fifth-instar nymphs. *Streptococcus* was enriched in various tissues of older nymphs, while *Pseudonocardia* and *Chloroplast* were enriched in the salivary glands of first-instar nymphs, and the remaining genera were enriched in the salivary glands (Figure 5B).

### 3.5. Functional Analysis of Bacterial Communities at Different Developmental Stages and in Various Tissues of R. dorsalis

**Figure 6:**
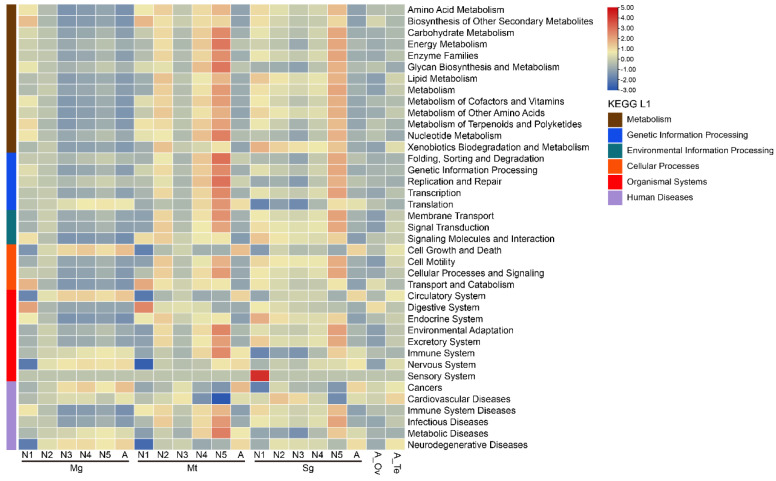
Comparison of KEGG prediction functions of core microorganisms in the midgut, salivary glands, Malpighian tubule, ovary, and testis of the leafhopper *R. dorsalis* at different developmental stages. N1, N2, N3, N4, N5, A refer to 1st−instar nymphs, 2nd−instar nymphs, 3rd−instar nymphs, 4th−instar nymphs, 5th−instar nymphs, and adults, respectively.

In order to confirm the different functional contributions of host-microbiota, we used PICRUSt2 to predict the relative abundance of KEGG level 2 functions related to the microbiome in all samples and analyzed the results by clustering heatmap. Microbiota functions in *R. dorsalis* mainly included Metabolism, Genetic Information Processing, Environmental Information Processing, Cellular Processes, Organismal Systems, and Human Diseases (Supplementary Table S5). Bacterial communities in first-instar nymphs were mainly clustered in cell growth and death, circulatory system, and neurodegenerative diseases. KEGG pathways gradually enriched with the development of *R. dorsalis* in the second to fifth-instar nymphs (Figure 6). Microbiota functions in the midgut were mainly enriched in cell growth and death, circulatory system, nervous system, and neurodegenerative diseases, while most KEGG level 2 functions in Malpighian tubules and salivary glands were enriched (Figure 6).

## 4. Discussion

The structure and abundance of microbial communities within insects are influenced by various factors, including the developmental stage of the insect host, dietary conditions, and environmental factors [20,21]. Our study shows that the developmental stage of *R. dorsalis* is the primary factor affecting bacterial community structure (Figure 3), which is consistent with many previous studies on Diptera, Lepidoptera, Coleoptera, and Hemiptera [22,23,24,25,26,27,28,29,30]. The bacterial community diversity in *R. dorsalis* decreased from the second instar, and a core bacterial community gradually forms (Figure 3). Similarly, in *Riptortus pedestris* and *Pyrrhocoris apterus*, the core bacterial community was successfully established in the second instar [28,45]. In addition, during the gradual formation of *R. dorsalis*’ core bacterial community, its function enriched gradually towards KEGG metabolism-related pathways (Figure 6). This suggests that the core bacterial community is primarily responsible for aiding in the digestion and nutrient supply of insect food. As newly hatched nymphs have a large amount of nutrients in their gut that can support development to the second instar without feeding, the core microbial community may not be necessary at this early stage [45]. As a result, the core bacterial community gradually forms after the insects start feeding.

The KEGG functional prediction related to microbial communities showed that the bacterial community within *R. dorsalis* is involved in various insect life activities and may also be related to various human diseases. In humans and mammals, gut microbiota imbalance has been associated with the pathogenesis of chronic non-communicable diseases, ranging from cardiovascular, neurologic, respiratory, and metabolic illnesses to cancer [46]. However, due to the complexity of the mammalian gut microbiome, only a few potential mechanisms of interaction between human diseases and gut microbiota have been explored [46]. For example, studies on human neurodegenerative diseases have found that the abundance of *Lactobacillus brevis* and *Bifidobacterium dentium* in the gut of Alzheimer’s disease (AD) patients is reduced [47]. Similar phenomena have also been found in Drosophila AD models, where the abundance of *Acetobacter* and *Lactobacillus* in the gut of Drosophila AD models is reduced [48]. Supplementing specific Lactobacillus and Acetobacter can partially restore the phenotype of the AD model fruit fly gut [48]. Subsequent studies have shown that serotonin synthesized and secreted by gut bacteria and the neurotoxin β-N-methylamino-L-alanine (BMAA) play an important role in the development of AD [47,49,50,51]. The secretions of microbial communities within various animal bodies may be related to the occurrence and development of many diseases. The stability of microbial communities within various animal bodies may be related to their health.

Differences in microbiota composition and abundance among the midgut, Malpighian tubules, and salivary glands of *R. dorsalis* may be related to its feeding behavior and food sources. Previous studies on liquid-feeding insects with sucking mouthparts, such as mosquitoes and ticks, have found that the diversity of microbial communities in the salivary glands is higher than that in the midgut [52,53,54]. This may be attributed to the probing behavior of sucking insects, which repeatedly detect potential feeding sites and continuously absorb fluid around their mouthparts to evaluate their suitability for feeding [55,56]. During this process, surface microbiota may be introduced into the salivary glands, leading to increased microbiota diversity (Figure 3 and Figure 4). Moreover, a study of bamboo-feeding insects revealed that solid-feeding insects with chewing mouthparts had more diverse gut microbiota compared to liquid-feeding insects with sucking mouthparts [57]. This may be due to the complexity of leaves and shoots compared to sap, which leads to an increase in the microbial content of the food source, and insects require more microbes to aid in digestion and absorption [58]. Liquid diets, such as animal or human blood and plant sap, are generally considered to be sterile or contain only a few microbes [59], potentially explaining why the midgut bacterial community diversity of *R. dorsalis* is low and stable throughout its life cycle (Figure 3 and Figure 4).

Proteobacteria is the most abundant phylum in the entire life cycle of *R. dorsalis*, except for the first-instar nymph and ovaries. In the study of Hemipteran, Proteobacteria is the most predominant bacterial phylum [60,61], possibly due to its involvement in host adaptation, digestion, nutrition supply, and energy metabolism [62]. The *Tistrella* bacteria is the most abundant bacteria in *R. dorsalis* during the period from the second instar to adult insects, accounting for over 80% of relative abundance. Research on the *Tistrella* genus of bacteria is limited, but a *Tistrella mobilis* strain isolated from the ocean can synthesize the antitumor compound didemnin B [63]. Additionally, a *Tistrella* sp. strain isolated from soil may have the ability to detoxify permethrin [64]. In *R. dorsalis*, the *Tistrella* genus of bacteria rapidly accumulates after the first feeding of the nymph, and it is speculated that they may be related to food digestion and host nutritional supply. Additionally, bacteria of the genus *Tistrella* may be vertically transmitted in *R. dorsalis*, but unlike the obligate symbionts *Sulcia* and *Nasuia* in leafhoppers [33], which have a lower relative abundance in the ovaries and first-instar larvae, the *Tistrella* genus bacteria have a higher relative abundance in the midgut of the first-instar nymph than in the Malpighian tubules and salivary gland, suggesting that they may be vertically transmitted by attachment to the egg surface, and the hatched nymph then consumes the eggshell to introduce them into the body.

The *Pantoea* genus bacteria are the second most abundant bacteria in various tissues of *R. dorsalis*. *Pantoea* bacteria are widely distributed in various environments and can exhibit both beneficial and harmful behaviors [65]. In insects, *Pantoea* colonizes the insect gut and plays a role in protein hydrolysis, pathogen resistance, toxin degradation, nitrogen fixation, and nutrient digestion [65]. In this study, *Pantoea* was found to be the most abundant bacteria in the Malpighian tubules of *R. dorsalis* fifth-instar larvae. The Malpighian tubules are the primary excretory organs of leafhoppers, responsible for the formation of primary urine and the elimination of metabolic waste [66]. Our unpublished study has shown that *Pantoea* bacteria in *R. dorsalis* play a crucial role in the recycling of nitrogenous waste. Therefore, we hypothesize that the gradual accumulation of *Pantoea* bacteria in the Malpighian tubules of *R. dorsalis* older nymphs may be related to the nitrogenous nutrient supply of *R. dorsalis.*

The microbiota of the first-instar nymphs of *R. dorsalis* strongly resembles that in adult female ovaries (Figure 4), indicating that the initial microbiota in *R. dorsalis* is mainly derived from the vertical transmission of parents through the ovary. *Sulcia* and *Nasuia* are two obligate symbiotic bacteria in *R. dorsalis*, which are also the most abundant bacteria in the midgut and Malpighian tubules of the first-instar nymphs and ovaries of adult females (Figure 5B). Previous studies have generally believed that the obligate symbiotic bacteria of leafhopper are restricted to bacteriocytes of mesodermal origin [67]. However, our study found that *Sulcia* and *Nasuia* were present in the gut, Malpighian tubules, and salivary glands of *R. dorsalis*, and a similar phenomenon was observed in the cicada *Subpsaltria yangi* [68]. In addition, our data showed that the bacterial community structure and abundance in the salivary glands of the first instar nymphs differed from those in the midgut and Malpighian tubules (Figure 4), which may be related to their different embryonic origins, with the midgut and Malpighian tubules developing from the endoderm and the salivary glands developing from the ectoderm [69].

In summary, we investigated the bacterial community of *R. dorsalis* throughout its entire life cycle in different tissues. The results indicated that the initial microbiota in *R. dorsalis* mostly originated from vertical transmission via the ovaries of the parents. After the first meal, the diversity of the microbiota decreased gradually, and a core bacterial community formed. We analyzed the core bacterial community and predicted its function, which mainly contributed to food digestion and nutrient supply. Although our study provides preliminary insights through bioinformatics analysis, further research is needed to understand the specific process of vertical transmission and verify the exact biological functions of the core microbiome. The comprehensive characterization of the bacterial communities associated with leafhopper *R. dorsalis* achieved in this study will serve as a foundation for future investigations into the development of novel biocontrol strategies against *R. dorsalis*, leveraging the intricate interactions between insects and their symbiotic microorganisms.

## Data Availability

The raw Illumina sequences generated for this study can be found in China National Microbiology Data Center (NMDC) under BioProject number NMDC10018402 (https://nmdc.cn/resource/genomics/project/detail/NMDC10018402).

## Supplementary Materials

The following supporting information can be downloaded at: https://www.mdpi.com/article/10.3390/insects14060545/s1, Figure S1. Relative abundance of 12 ASVs present in all samples in different tissues of leafhopper *R. dorsalis* at different developmental stages; Figure S2. Rarefaction curves showing bacterial richness based on the Chao1 index and bacterial richness and evenness on the Shannon index; Figure S3. Analysis of alpha diversity among different tissues of leafhoppers at the same developmental stage; Table S1. Statistics of the sequencing data preprocessing and quality control; Table S2. Statistical results of ASV characteristic sequences of each sample; Table S3: Microbial community alpha-diversity characteristics at different developmental stages and in various tissues of *R. dorsalis*; Table S4. The LEfSe analysis revealed significant differential of bacterial species at the phylum, class, order, family, and genus levels; Table S5: Comparison of KEGG prediction functions of core microorganisms in the midgut, salivary glands, Malpighian tubule, ovary, and testis of the leafhopper *R. dorsalis* at different developmental stages.
