# Supplementary material for: Diversity and Dynamics of Bacterial Communities in the Digestive and Excretory Systems across the Life Cycle of Leafhopper, Recilia dorsalis
Source: Insects. 2023 Jun 12;14(6):545. doi: 10.3390/insects14060545 (PMC10299453; doi:10.3390/insects14060545)
Supplement: Supplementary file 1 [file insects-14-00545-s001.zip › insects-2394396-supplementary/Supplementary Figures.pdf]

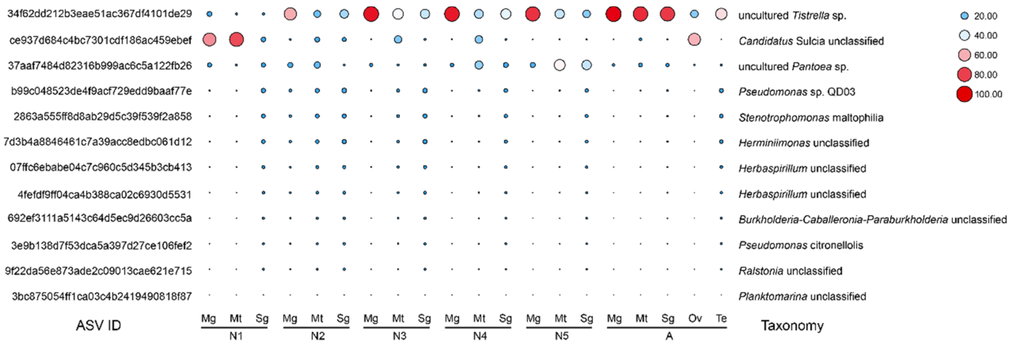

Figure S1. Relative abundance of 12 ASVs present in all samples in different tissues of leafhopper *R. dorsalis* at different developmental stages. Circle area corresponds to relative abundance of bacteria in each sample. N1, N2, N3, N4, N5, A refer to 1st-instar nymphs, 2nd-instar nymphs, 3rd-instar nymphs,4th-instar nymphs, 5th-instar nymphs and adults, respectively. Mg, midgut; Mt, Malpighian tubule; Sg, salivary gland.

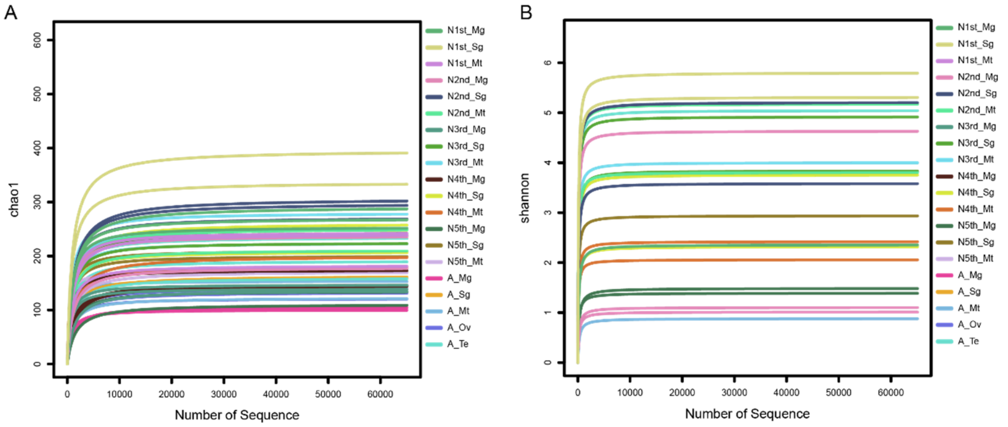

Figure S2. Rarefaction curves showing bacterial richness based on the Chao1 index (**A**) and bacterial richness and evenness on the Shannon index (**B**).

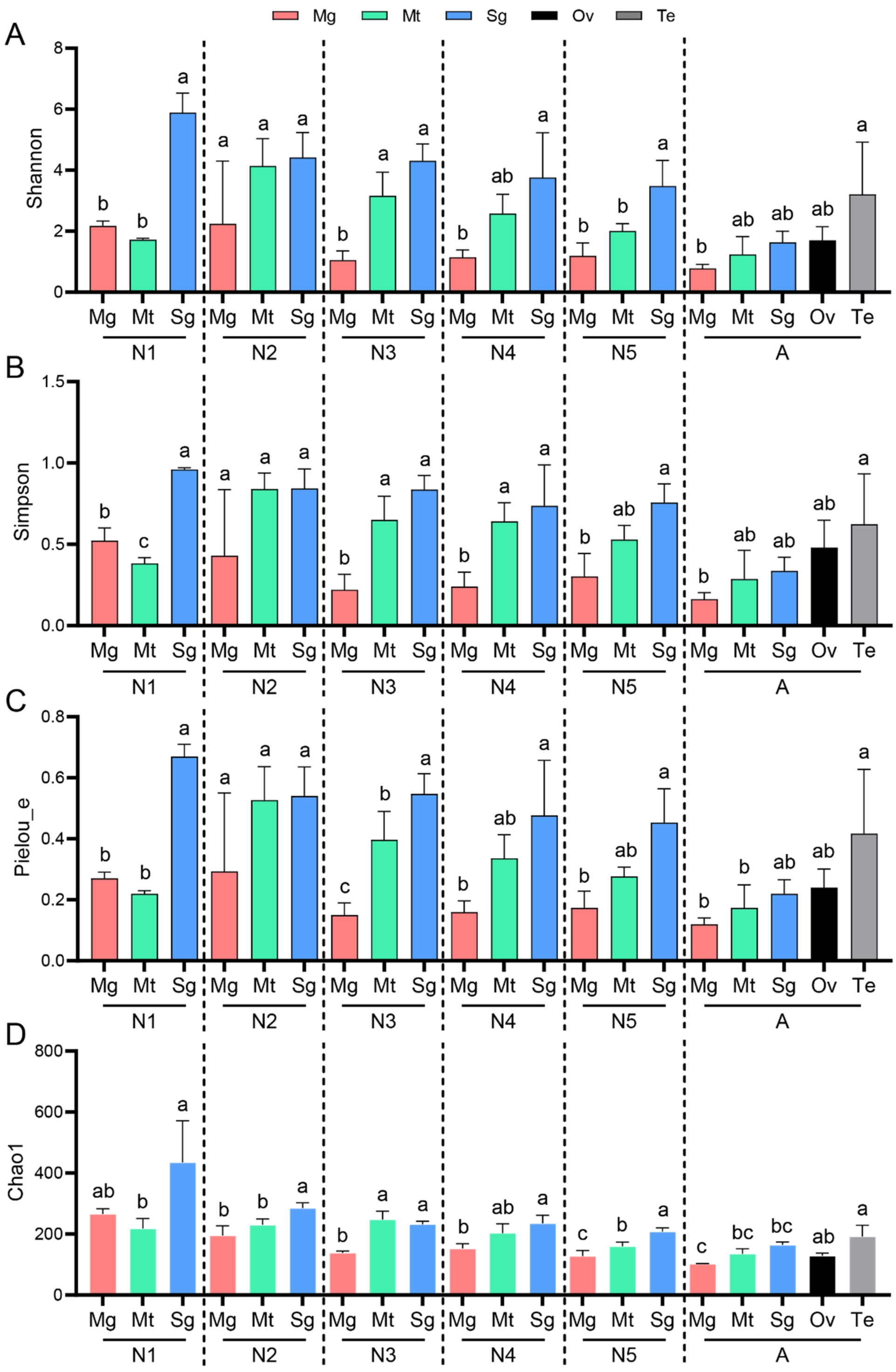

Figure S3. Analysis of alpha diversity among different tissues of leafhoppers at the same developmental stage. Shannon (**A**) and Simpson (**B**) indexes represent the species diversity, Pielou_e (**C**) index represents species evenness, Chao1 (**D**) species indexes represent species richness. Each bar represents the mean ± standard error of three replicates. Different lowercase letters indicate significant differences (ANOVA, $p < 0.05$). N1, N2, N3, N4, N5, A refer to 1st-instar nymphs, 2nd-instar nymphs, 3rd-instar nymphs,4th-instar nymphs, 5th-instar nymphs and adults, respectively.
